# Redox activation of ATG5 licenses autophagy upon nutrient restriction

**DOI:** 10.64898/2026.09.23.753803

**Published:** 2026-09-24

**Authors:** Sanghee Shin, Ekaterina D. Korobkina, Claudia Lennicke, Nils Burger, Bingsen Zhang, Haopeng Xiao, Yaoyu Wang, Jonathan J. Petrocelli, Shelley M. Wei, David Wood, Yu Lei, Xiaomei Zhang, Maria C. Perez-Matos, Muneeb A. Sharif, Jiuchun Zhang, Helena M. Cochemé, William B. Mair, Edward T. Chouchani

## Abstract

Dietary restriction (DR) protects against metabolic disease, extends lifespan, and is associated with remodeling of tissue reactive oxygen species (ROS). ROS control biological adaptation through reversible oxidation of protein cysteines, yet the targets of DR-initiated redox signaling are unknown. Here we generate OxiDR, a tissue-resolved atlas of the cysteine redox proteome that quantifies oxidation state under DR. Rather than oxidizing the proteome broadly, DR selectively targets a high-amplitude set of cysteines in a tissue-specific manner, allowing systematic classification of biological processes subject to DR-mediated redox regulation. Among the cysteines most highly oxidized upon DR is Cys19 of the core autophagy protein ATG5. We show oxidation of Cys19 is required for ATG5-mediated autophagosome formation and for autophagy triggered by nutrient restriction in human cells and mice. Reversible oxidation of this cysteine promotes ATG5 binding to ATG10, thus forming the ATG5–ATG12 conjugate that lipidates LC3B/ATG8 and matures the autophagosome. In mice, loss of this redox switch prevents effective initiation of autophagy upon nutrient restriction, resulting in gross tissue pathology and rapid onset of mortality. The autophagic response to nutrient restriction is thus gated by oxidation of a single cysteine.

## Full Text Availability

The license terms selected by the author(s) for this preprint version do not permit archiving in PMC. The full text is available from the preprint server.

